# Health-related quality of life measured by EQ-5D-3L for the spouses of breast cancer patients

**DOI:** 10.3389/fonc.2022.983704

**Published:** 2022-10-18

**Authors:** Li-Fei Sun, Sheng Huang, Yun-Fen Li, Zhuang-Qing Yang, Xiao-Juan Yang, Jie-Ya Zou, Xiao-Wen Wang, Jian-Yun Nie

**Affiliations:** ^1^ Breast Cancer Institute, The Third Affiliated Hospital of Kunming Medical University, Yunnan Cancer Hospital, Kunming, China; ^2^ Center for AIDS/STDs Prevention and Control, Yunnan Center for Disease Control and Prevention, Kunming, China

**Keywords:** breast cancer, spouse, health-related questionnaire, life quality, EQ-5D-3L

## Abstract

To explore factors influencing the health-related quality of life of spouses of breast cancer patients and the suitable questionnaires for this purpose. A cross-sectional study was conducted in the Third Affiliated Hospital of Kunming Medical University. The spouses of breast cancer patients were included and evaluated *via* face-to-face interviews. Self-designed demographic characteristics and disease-related questionnaires, the 12-item health survey questionnaire (SF-12), the three-level European five-dimensional health status scale (EQ-5D-3L), and the Social Support Rate Scale (SSRS) were used. The internal consistency reliability measure Cronbach’s coefficient, criterion-related validity, construct validity, and sensitivity were used to evaluate the applicability of the EQ-5D-3L. Univariate and multivariate analyses were performed to analyze the factors associated with the health-related quality of life of spouses of breast cancer patients. We investigated a total of 100 spouses of breast cancer patients. Cronbach’s α, the internal consistency reliability coefficient, was 0.502. The EQ-5D-3L health utility score was moderately correlated with PCS-12 (r=0.46, p=0.0001) and weakly correlated with MCS-12 (r=0.35, p=0.0001). The EQ-5D-3L health utility score for the spouses of breast cancer patients was 0.870, and the EQ-VAS was 78.3. In multivariate analysis, social support and cognition of the treatment effect were factors that influenced the EQ-5D-3L health utility score. The EQ-5D-3L has good reliability, validity, and sensitivity for measuring the physiological aspects of the health-related quality of life of spouses of BC patients. EQ-5D-3L was considered suitable for this study.

## Introduction

According to GLOBOCAN cancer statistics in 2021, breast cancer (BC) causes 24.5% of new cancer diagnoses among females and raised to the leading cancer-related cause of death in this group (1). The diagnosis of BC is a major psychological event for young BC patients. They face treatment for reproductive function, impacts on marital relationship, influence on young children’s education, appearance changes (such as hair loss due to chemotherapy), and secondary sexuality loss due to operation and fear of future career stability (2). BC not only seriously affects patient’s physical health and quality of life but also places serious financial and psychological burdens on the family. In China, For female BC patients, spouses are their main caregivers. Some studies have noted that when a married woman is diagnosed with a malignant tumor, her spouse is at higher risk of psychological distress (3). As co-sufferers and the most important supporters of BC patients, spouses may experience emotional disorders, stress, and impaired physiological functions which further aggravate the burden of care, ultimately adversely affecting patients’ treatment and quality of life and even causing marital crisis (4, 5). Both medical staff and patient family members focus on the treatment and quality of life of BC patients, but spouses are often neglected.

In the era of biopsychosocial medicine, increasing attention has been paid to the comprehensive health status of BC patients and their families (6). Health-related quality of life (HRQOL) (7) has been proposed to be a comprehensive reflection of physical health, mental health, and social and emotional aspects of modern health. Health-related quality of life is usually measured by scales. Therefore, the selection of appropriate measurement tools is an important step to accurately assess quality of life in the target population. The three-level Euro Quality of life five-dimension (EQ-5D-3L) survey and 12-item Short Form Health Survey (SF-12) are two widely used tools in health-related quality of life measurement. The SF-12 is a simplified version of the MOS 36-item short from the health survey (SF-36), and its reliability and validity have been demonstrated (8).

The EQ-5D-3L is a health measurement scale based on “single preference”. The survey results reflect the burden of disease, obtain a comprehensive score of the health level of the target population, and help health decision-makers assess reasonable allocation of health resources (9). Many studies to date have adopted the EQ-5D-3L scale to measure the quality of life of patients with diabetes, hypertension, coronary heart disease, and stroke (10). However, no research has been conducted on health-related quality of life of spouses of BC patients using the EQ-5D-3L scale.

This study used both EQ-5D-3L and SF-12 to assess the health-related quality of life for spouses of BC patients to provide evidence for the applicability of the EQ-5D-3L and to assess the health utility of this population. By analyzing the influencing factors, we obtained evidence to inform intervention strategies.

## Methods

This study was conducted at the Third Affiliated Hospital of Kunming Medical University in 2019, Yunnan Province. During the study period, about 520 patients were treated, and 80% of them were accompanied by parents, children, siblings, spouses and friends. About 36% of breast cancer patients are accompanied by their spouses, and some of them do not want to participate. This study is a cross-sectional study, and convenience samplingmethod was used to conduct the survey. From the perspective of statistics,100 samples could meet the needs of the study. All participants provided written informed consent. Data were collected by face-to-face interviews. For those caregivers and patients who were illiterate or had difficulty reading or writing the questionnaires, the researchers read the questions to them and recorded their responses. Demographic and clinical information of patients was confirmed by reviewing their medical records. The self-report survey consisted of two questionnaires that were separately completed by patients and spouse caregivers. Five parts of the survey were used for analysis and discussion: 1) spouse demographic characteristics, including date of birth, age at marriage, education level, current occupation, work status in the past three months, main economic income for family, type of medical insurance, support individuals’ level of knowledge about the illness, and whether they have chronic diseases; 2) European five-dimensional three-level health status scale (EQ-5D-3L); 3) social support rating scale (SSRS); 4) short form 12 health survey (SF-12); and 5) spouses’ knowledge of the patient’s disease, including the severity of BC, curability of the disease, survival time, treatment effect, treatment attitude, and economic burden. A cross-sectional design was applied to collect data from patients with BC and from their male spouse caregivers.

### Scales

#### EQ-5D-3L

The EQ-5D-3L is composed of two parts: a questionnaire and a utility value conversion table (11). Questionnaire results can be used to describe the health status of the population and obtain the visual analog scale (VAS) score. The EQ-5D-3L index score can be further obtained by using the utility value conversion table for the Chinese population[12]. The EQ-5D-3L scale includes 5 dimensions: mobility, self-care, usual activities, pain/discomfort, and anxiety/depression. Each dimension has 3 levels: no difficulties, some difficulties, and extreme difficulties. EQ-VAS is a visual scale with vertical isometric scales marked 0 and 100 at the top and bottom, respectively. We adopted the time trade-off (TTO) method to calculate it based on the scale utility score system obtained in the Chinese population (12, 13) (**Table 1**).

**Table 1:** EQ-5D-3L adopted the Time trade-off by Chinese population.

Dimensions	No problem	Some problems	Extreme problems
Constant term	0.039	–	–
Mobility	0.000	0.099	0.246
Self-care	0.000	0.105	0.208
Usual activities	0.000	0.074	0.193
Pain/discomfort	0.000	0.092	0.236
Anxiety/depression	0.000	0.086	0.205
N3	0.022	–	–

#### SSRS

The Social Support Rate Scale (SSRS) was established by the Xiao Shui Yuan in 1986 and includes three dimensions and ten items. The three dimensions are objective social support, subjective social support, and support utilization. Social support can be divided into three dimensions: objective social support, subjective social support, and support utilization.

#### SF-12

This scale includes 8 dimensions and 12 items in total[9][10]. The 8 dimensions are Physical Functioning (PF), Role-physical (RP), Bodily Pain (BP), General Health (GH), Vitality (VT), Social Functioning (SF), Role-Emotional (RE), and Mental Health (MH). GH, PF, RP, and BP are included in the physical component summary (PCS). RE, MH, VT, and SF are included in the mental component summary (MCS). Scores of SF-12 were calculated using the standard scoring method of SF-12 (2nd edition) in the United States.

### Data analysis

Epidate 3.1 software was used for duplicate entry of the questionnaire results to ensure correct data entry. The SPSS 19.0 statistical software package was used for data sorting and statistical analysis. Descriptive statistics used to show the demographic characteristics of the study sample. The applicability of EQ-5D-3L scale was analyzed with Cronbach’s alpha coefficient of internal consistency reliability. Sensitivity and structural validity analysis mainly includes two parts: the sensitivity of EQ-5D health utility scores and EQ-VAS scores and an assessment of the correlations between EQ-5D and SF-12 component scores. In analysing the discrimination ability of EQ-5D health utility scores and EQ-VAS scores, the key point is whether EQ-5D health utility scores and EQ-VAS scores are sensitive enough to discriminate the differences between cut-off scores of PCS-12 and MCS-12, respectively. First, respondents were divided into two groups by the mean scores of PCS-12 and MCS-12 at the median or lower (SF-12 ≤ median) and higher than mean scores (SF-12 > median). In addition, we compared mean EQ-5D health utility scores and mean EQ-VAS scores across this category to infer discrimination ability of EQ-5D health utility scores and EQ-VAS scores, respectively, We used a multitrait-multimethod (MTMM) matrix to assess the structural validity between EQ-5D and SF-12 component scores by Pearson correlation analysis.The correlation coefficient can be defined at five levels: 1 is perfect, 0.7 to 0.9 is strong, 0.4 to 0.69 is moderate, 0.1 to 0.39 is weak and 0 is no correlation (14).

All the EQ-5D health utility scores and EQ-VAS scores are quantitative data. One-way ANOVA was used for univariate analysis. Multiple linear regression models with the EQ-5D health utility score and EQ-VAS score as dependent variables were constructed for multivariate analysis, and ordinary least squares (OLS) was used for parameter estimation. The standard for including model variables in univariate analysis was *P*<0.1. The significance level was set as α=0.05.

## Results

### Spouse demographic characteristics of BC patients

A total of 100 spouses of BC patients were investigated in this study, with an average age of 48.23 ± 9.52 years. The education level was mainly middle school level, accounting for 32% of subjects; the occupations were mainly farmer and migrant worker, accounting for 42% of subjects. Marital status was mainly first marriage, accounting for 96% of subjects. The types of medical insurance were mainly residents’ medical insurance, accounting for 54% of subjects. The stage of their spouses’ BC was mainly stage II, accounting for 61% of subjects In this study, the social support score was 45.4 ± 6.16.

### Measurement results, reliability, validity, and sensitivity analysis of EQ-5D-3L

#### Measurement results of EQ-5D-3L

**Figure 1:**
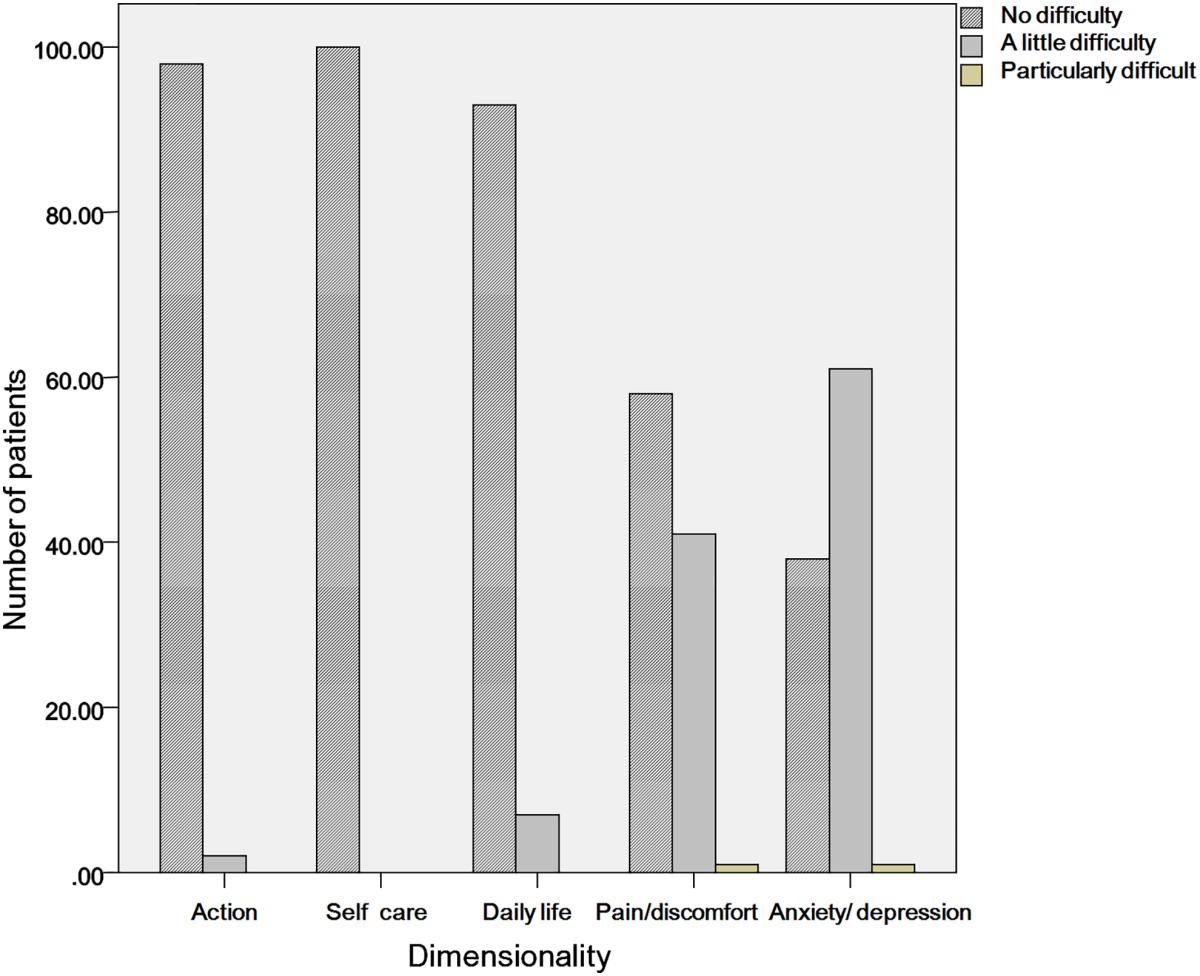
The distribution of the EQ-5D scores.

In the measurement of health-related quality of life, the maximum EQ-5D-3L health utility score was 1.000, the minimum score was 0.469, and the median EQ-5D-3L health utility score was 0.875. The interquartile range was 0.21, the mean health utility score was 0.870, and the standard deviation was 0.106. The median EQ-VAS score was 80.0, the interquartile range was 20.0, the mean score was 78.3, and the standard deviation was 12.2. The distribution of EQ-5D-3L and EQ-VAS measurement results is shown in **Table 2** and **Figure 1**.

**Table 2:** Distribution of EQ-5D-3L measurement results.

Combination method	Number of cases	Percentage (%)	Cumulative percentage percentage (%)
11111	28	28	28
11112	30	30	58
11121	10	10	68
11122	25	25	93
11222	4	4	97
11232	1	1	98
21222	1	1	99
21223	1	1	100
Total	100	100	–

#### Reliability of EQ-5D-3L

This study was a cross-sectional survey. Only one questionnaire survey was conducted, and reliability of the duplicate reliability and the test-retest reliability were not applicable. From a practical view, the half-fold reliability is economical and simple. However, there are still some shortcomings. At present, there is no theoretical deduction to rigorously prove its validity. Second, for the same set of problems, there may be multiple combinations, which leads to the calculation of the half-fold reliability with some randomness. In this study, the internal consistency reliability was evaluated using Cronbach’s coefficient method (Cronbach’s α coefficient method). The Cronbach’s α internal consistency reliability coefficient calculated in this study was 0.502.

#### Validity of EQ-5D-3L

The EQ-5D-3L has been widely used in many diseases, and the scale has good content validity. This study mainly analyzed the criterion-related validity and construct validity. In the constructed MTMM (Multiple Trait Multiple Method) matrix, Pearson correlation analysis showed that the EQ-5D-3L health utility score was moderately correlated with PCS-12 (r=0.46, p=0.0001) and was weakly correlated with MCS-12 (r=0.35, p= 0.0001), EQ-VAS score was weakly correlated with PCS-12 (r=0.37, P=0.0001) and MCS-12 (r=0.29, p=0.004), and the EQ-5D-3L health utility score and the EQ-VAS score had a moderate correlation (r = 0.40, p=0.0001) (**Table 3**).

**Table 3:** MTMM matrix analysis Correlation between EQ-5D-3L and SF-12 scores.

	SF-12	SF-12	EQ-5D	EQ-5D
	(PCS-12 Score)	(MCS-12 Score)	(Health utility score)	(EQ-VAS Score)
SF-12(PCS-12 Score)	1	–	–	–
SF-12(MCS-12 Score)	0.49*	1	–	–
EQ-5D (Health utility score)	0.46*	0.35*	1	–
EQ-5D (EQ-VAS Score)	0.37*	0.29*	0.40*	1

*P<0.001

The EQ-5D-3L includes both physical and psychological measurements. Self-illumination, daily activities, and pain/discomfort mainly reflect physiological measurements; anxiety/depression mainly reflects psychological measures. The PCS-12 scores were lower for respondents who reported a “problem” in the EQ-5D-3L action, daily activities, pain/discomfort, or anxiety/depression dimensions. “Problems” were reported for EQ-5D-3L in the pain/discomfort and anxiety/depression dimensions, and these subjects had a lower MCS-12 score. From the analysis of the validity of polymerization, the physiological function reported by EQ-5D-3L had a stronger correlation with PCS-12. The psychological function reported by EQ-5D-3L had a stronger correlation with MCS-12. From the analysis of discriminant validity, the EQ-5D-3L and SF-12 had weak or no correlations in the measurement of different traits (**Table 4**).

**Table 4:** Comparison of SF-12 scores in different dimensions of EQ-5D-3L.

EQ-5D-3LDimension	Level	N	PCS-12			MCS-12		
			$\bar{X}$ ± S	P*	η-sqa	$\bar{X}$ ± S	P*	η-sqa
action	1	98	48.43 ± 10.01	0.02	0.06	50.05 ± 8.69	0.11	0.003
	2,3	2	31.32 ± 5.05	–	–	43.68 ± 8.62	–	–
Self-care	1	100	48.09 ± 10.22	–	–	49.85 ± 8.75	–	–
	2,3	0	–	–	–	–	–	–
Daily activities	1	93	49.40 ± 9.13	0.001	0.22	50.71 ± 7.69	0.001	0.13
	2,3	7	30.60 ± 7.94	–	–	38.42 ± 13.95	–	–
Pain/ discomfort	1	58	50.30 ± 6.67	0.01	0.07	51.30 ± 6.67	0.05	0.04
	2,3	42	45.03 ± 12.13	–	–	47.85 ± 10.78	–	–
Anxiety/ depression	1	38	52.58 ± 5.53	0.0003	0.09	53.15 ± 5.52	0.001	0.12
	2,3	62	45.33 ± 11.43	–	–	47.83 ± 9.74	–	–

*SF-12 score by One-way ANOVA.

a η-sq =ssmodel/sstotal Indicates the strength of the correlation between variables after controlling the effect of sample content in the analysis of variance.

#### Sensitivity

In this study, EQ-5D-3L sensitivity was measured by comparing the EQ-5D-3L health utility scores and the EQ-VAS scores that were sensitive enough to detect differences in the different health states defined by the SF-12 score. First, the median scores of the PCS-12 and MCS-12 were used as cut-off points. According to the scores equal to or lower than the median and greater than the median, the subjects were divided into two groups, and the EQ-5D-3L scores were compared with each group. The difference between the EQ-5D-3L health utility score and the EQ-VAS score was used to evaluate the different health statuses of the spouses of BC patients.

Using the median PCS-12 and MCS-12 scores as the cut-off points, the subjects were divided into two groups. The EQ-5D-3L health utility score and EQ-VAS score in the median array were lower than the PCS-12 score and higher than the median array. There was no significant difference in the EQ-5D-3L health utility score and EQ-VAS score between MCS-12 equal to or lower than the median array and MCS-12 above the median array (t=-0.9943, P=0.3225) (**Table 5**).

**Table 5:** Comparison of EQ-5D-3L scores by PCS-12 and MCS-12 median grouping.

SF-12	Cutoff point	N	EQ-5D-3L health utility score ( $\bar{X}$ ± S)	P	EQ-VAS $\bar{X}$ ± S	P
PCS-12	≤46.47	53	0.84 ± 0.11	0.003	74.53 ± 12.64	0.0009
	>46.47	47	0.90 ± 0.08	–	82.45 ± 10.21	–
MCS-12	≤52.35	82	0.86 ± 0.11	0.2981	77.68 ± 12.48	0.3225
	>52.35	18	0.89 ± 0.02	–	80.83 ± 10.61	–

The EQ-VAS scores of 28 subjects who were in the “best health status” (“no problem” reported in five dimensions) in the EQ-5D-3L measurement were truncated according to the median of PCS-12 and MCS-12. The EQ-VAS scores of PCS-12 below the median array were lower than the median array of PCS-12, and the EQ-VAS score and MCS-12 were lower than the median array. The difference between MCS-12 equal to or higher than the median array was statistically significant (t=-2.3985, P=0.0239) (**Table 6**).

**Table 6:** Comparison of EQ-VAS scores for the “best health status” respondents by PCS-12 and MCS-12 median grouping.

SF-12	Cutoff point	N	EQ-VAS( $\bar{X}$ ± S)	P
PCS-12	<56.57	10	78.00 ± 12.06	0.0239
	≥56.57	18	87.22 ± 8.26	–
MCS-12	<52.35	21	84.52 ± 11.17	0.5089
	≥52.35	8	82.14 ± 9.06	–

### Univariate analysis of factors affecting the quality of life of spouses in patients with BC

One-way analysis of variance (one-way ANOVA) was used for univariate analysis of factors affecting quality of life. The analysis showed that the EQ-5D-3L health utility score was in BC staging (F=3.24, p=0.03), whether BC could be cured (F=3.96, p=0.02), the comprehensive treatment effect of BC (F =9.22, p=0.001), treatment cost and stress (F=3.93, p=0.02), and social support (F=8.14, p=0.01). The difference was statistically significant (p<0.05), showing that early staging, BC can be cured, the treatment is considered to be ineffective, treatment cost is low, and social support scores were higher. The group had a higher EQ-5D-3L health utility score. EQ-VAS scores were as follows: combined chronic disease (F = 4.11, p= 0.02), maternal BC staging (F = 2.75, p= 0.047), considered the comprehensive treatment effect of BC (F = 6.42, p= 0.002) and society support (F=5.33, p=0.02). The difference was statistically significant (p<0.05) and was characterized by early maternal staging, no confirmed chronic disease, and higher scores in the group with higher comprehensive treatment and social support scores.

### Multivariate analysis of factors affecting the quality of life of spouses in patients with BC

This study used a general linear regression model to analyze the factors affecting quality of life. After controlling for the influence of other related factors, social support and cognition of the BC treatment effect are factors that influence the EQ-5D-3L health utility score. The higher the social support score, the higher the health utility score. Additionally, subjects who were considered treatment effective were less likely to have a higher EQ-5D-3L health utility score than those who did not. The family’s per capita annual income level and cognitive treatment of BC influenced EQ-VAS score. The household per capita annual income level in the 50,00-10,000 RMB group had a lower EQ-VAS score than the ≤5,000 RMB group. The respondents who considered treatment effective were less likely to have a higher EQ-VAS score than those who were aware of the treatment effect (**Table 7**).

**Table 7:** Analysis of factors affecting the quality of life of spouses in patients with breast cancer.

Variable	EQ-5D Health utility score				EQ-VAS Score			
	Coef.	SE	t	95%CI	Coef.	SE	t	95%CI
Social support score ^*^	0.004	0.002	2.300	(0.001,0.007)	0.328	0.194	1.690	(-0.058,0.713)
Age (≤44)^a^								
44-59	–	–	–	–	4.533	2.541	1.780	(-0.523,9.589)
≥60	–	–	–	–	1.795	4.158	0.430	(-6.477,10.068)
Marital status (first marriage)^a^	–	–	–	–				
remarry	0.084	0.050	1.700	(-0.014,0.183)				
Whether you have chronic disease (yes)^a^								
no	–	–	–	–	4.122	3.064	1.350	(-1.973, 10.218)
unclear	–	–	–	–	7.504	4.745	1.580	(-1.973,10.218)
Spouse breast cancer staging (Stage I)^a^								
Stage II	0.035	0.024	1.480	(-0.001,0.101)	2.533	3.015	0.840	(-3.467,8.532)
Stage III	0.021	0.033	0.064	(-0.044,0.087)	-1.126	3.980	-0.280	(-9.045,6.794)
Stage IV	0.006	0.053	0.120	(-0.099,0.113)	-8.343	6.340	-1.320	(-20.957,4.272)
Think breast cancer is cured(unknown)^a^								
Yes	0.035	0.024	1.480	(-0.012,0.083)	1.578	2.784	0.570	(-3.962,7.118)
No	0.009	0.044	0.210	(-0.012,0.083)	-1.338	5.233	-0.260	(-11.750,9.074)
Life span (unknown)^a^								
1-5 years	-0.082	0.050	-1.660	(-0.181,0.016)	-4.348	6.004	-0.720	(-16.294,7.599)
5-10 years	-0.027	0.033	-0.820	(-0.091,0.038)	-2.644	3.859	-0.690	(-10.322,5.034)
More than 10 years	-0.012	0.022	-0.540	(-0.057,0.033)	1.151	2.778	0.410	(-4.376,6.677)
Treatment effect								
Useful^*^	0.057	0.026	2.180	(0.005,0.109)	7.056	3.106	2.270	(0.876,13.236)
Useless	0.090	0.071	1.280	(-0.050,0.231)	9.611	8.363	1.150	(-7.029,26.251)
No pressure about treatment fee^a^								
Expensive, stressful	0.017	0.029	0.580	(-0.041,0.074)	–	–	–	–
Expensive, a lot of stressful	-0.009	0.033	-0.260	(-0.073,0.056)	–	–	–	–
Annual income per person (≤5,000 RMB)^a^								
5,000-10,000 RMB	–	–	–	–	-5.961	4.060	-1.470	(-14.039,2.116)
10,000-50,000 RMB^*^	–	–	–	–	-8.969	3.367	-2.660	(-15.669,-2.269)
≥50,000 RMB	–	–	–	–	-5.170	4.276	-1.210	(-13.677,3.337)
Intercept ^*^	0.587	0.086	6.850	(0.416,0.757)	56.961	9.155	6.220	(38.745,75.176)

*P<0.05. ^a^Comparison groups.

## Discussion

The study confirmed the acceptable reliability and good validity, and sensitivity of the EQ-5D-3L scale in measuring the quality of life for the spouses of BC patients. Generally, the value of Cronbach’s α was more than 0.7 for a scale with 10 items, we could consider to be an acceptable level (15). However, EQ-5D-3L has only 5 items, the value of Cronbach’s α was usual less than the scales with more items. We consider it at an acceptable level. The correlation of the EQ-5D and SF-12 was illustrated by a multitrait-multimethod (MTMM) matrix. EQ-5D health utility scores were moderately correlated with both SF-12 components scores. A MTMM matrix is generally used to evaluate construct validity of measures (16). If we defined SF-12 as the “gold standard”, the construct validity could be identified for EQ-5D. Otherwise, a relatively stronger relationship was observed with the PCS-12, which indicated that the respective constructs of EQ-5D may not overlap and that our respondents gave more weight to their physical health when they provided a total health rating.EQ-5D health utility scores and EQ-VAS scores were also sensitive enough to discriminate the differences between the cut-off scores of the PCS-12.

The measurement results were authentic, and the scale is suitable for evaluating the quality of life in the spouses of BC patients. In previous studies, some scholars used the SF-12 and EQ-5D-3L scales to compare the applicability of life quality assessment in stroke and diabetes, respectively. The EQ-5D-3L scale applicability has been confirmed. Previous studies on the quality of life of BC patients used the Concise Health Status Questionnaire (SF-36) (17), Connor and Davidson Resilience Scale (CD-RISC) (18), Self-rating Anxiety Scale (SAS) (19), Self-rating Depression Scale (SDS) (20), and Symptom Checklist (SCL-90) (21). This study adds valid evidence supporting the applicability of the EQ-5D-3L scale in different populations. Additionally, the EQ-5D-3L has 5 dimensions and is simpler than the SF-12. The respondents have forced-choice options which is more conducive to on-site operation. In terms of the expression of measurement results, EQ-5D-3L yields a comprehensive health score, unlike SF-12. Since SF-12 cannot be directly used to assess health weights in health economics analysis, EQ-5D-3L can determine the weight of health and assess disease burden, which is more advantageous in supporting health decision making (22).

In this study, the highest score of the EQ-5D-3L health utility score was 1.000, and the lowest score was 0.469. The median EQ-5D-3L health utility score was 0.875, and the interquartile range was 0.217. The mean EQ-5D-3L health utility score was 0.870, and the standard deviation was 0.106. The EQ-VAS score was 100 points, and the lowest score was 40 points. The median EQ-VAS score was 80.0 points, the interquartile range was 20.0 points, the mean was 78.3 points, and the standard deviation was 12.2 points. The results of the study showed that the quality of life of the spouse of BC patients was worse than that of normal men (the utility score was 1 for normal healthy males), consistent with previous studies (23, 24). The quality of life for males with (10) diabetes was 0.79~0.94, hypertension was 0.78~0.93, coronary heart disease was 0.75~0.90, and chronic obstructive pneumonia was 0.64~0.80. From the perspective of health-related quality of life, being a spouse of a BC patient is equivalent to the disease burden faced by male hypertensive patients and diabetic patients. The quality of life of spouses in BC patients is an important part of evaluating the burden of BC disease. In the process of coping with the burden of malignant tumors, it should be considered by public health-related departments.

Since health-related quality of life reflects physical health, mental health, modern social and emotional health conditions, and both the disease state and also the psychological and social functional effects of the disease (25), health-related quality of life is affected by many factors (26). This study further analyzed the factors affecting the health-related quality of life of BC patients’ spouses and found that the social support and cognitive factors of treatment effects were influencing factors. In this study, the spouses of patients with higher social support scores had higher EQ-5D-3L health utility scores. Conversely, spouses with low social support scores had lower EQ-5D-3L health utility scores. Social support is an important external resource that individuals can use when dealing with stress, and it can reduce the negative impact of stressful events on personal well-being (27). In the treatment of malignant tumors, good social support not only improves the individual’s cognition during stress but also reduces the damage the individual faces during the stressful event and alleviates the physical and mental stress of the malignant tumor in patients and their spouses. It can also enhance family coping ability and improve quality of life (28, 29). Existing studies have confirmed that Chinese men are often silent about their stress and family problems (30). As a spouse of BC patients they serve as the primary caregiver, spending much time and energy to care for patients and handling daily work and family affairs while being influenced by traditional culture and social environments (31). Their incidence of mental illness is greater than or equal to that of cancer patients (32). Social support is often a contributing factor on the impact of psychological stress on quality of life (33). Therefore, spouses should be encouraged to mobilize family support, regardless of daily life, economic aspects, psychological support and comfort, and actively seek the care and help of other family members and friends; at the same time, it is necessary to formulate relevant support policies, such as community and work units for cancer patients. Additionally, the family supports the financial well-being so that the spouses of BC patients have more confidence in the fight against cancer.

Additionally, this study found that the perception of the treatment effect of BC patients’ spouses affects their health-related quality of life. Spouse of breast cancer patients with good BC treatment have a better quality of life, and those with unknown therapeutic effects have a worse quality of life. This is because in China, especially in rural areas, disease knowledge is insufficient, especially for malignant tumors, and individuals seldom “talk about cancer.” Yunnan Province is located in the western region of China and has many ethnic minorities. The regional economy (34), knowledge level, and cultural differences (35) are quite variable, and an understanding of malignant tumors is lacking. BC, even a malignant tumor with good clinical therapeutic effects, has cognitive biases in the perceived treatment effect (36). Some patients have undergone radical mastectomy because of the initial diagnosis of small tumors, and the patient’s spouse cannot be directly understood. The effects of subsequent chemotherapy and radiotherapy on BC may increase the psychological burden of the patient’s spouse and affect their quality of life. Therefore, the medical staff should fully communicate with the patient, their spouse, and other family members during the treatment. Public health agencies can organize popular science activities and fully explain the disease. Public health agencies should strengthen health education on BC-related knowledge and establish a correct understanding of BC.

This study was the first to use the EQ-5D-3L scale to assess the health-related quality of life of BC patients’ spouses and to explore the influencing factors.

There are some limitations in this study: the convenient sampling method is adopted in this study, and the sample source is limited to the patients in our hospital and department. The coverage is small, and the sample size is relatively small, which cannot fully represent the breast cancer spouses in China. Therefore, the results of this study cannot be generalized to the whole. This study is a cross-sectional study. The health-related quality of life of the surveyed population may change over time. In the future, we can try to carry out longitudinal studies. We will consider expanding the survey scope and sample size to obtain more data support. We could not calculate the test-retest reliability. We should study the test-retest reliability of EQ-5D in the BC spouse in the future research so that the accessibility could be verified with strong evidence. There is still a ceiling effect in our measurement. In our study, the proportion responding ‘no problem’ on each of the EQ-5D dimensions (self-reported health status ‘11111’) was 28%, which was higher than the average proportion of each health status (1/243 = 0.4%).

## Conclusion

The EQ-5D-3L has good reliability, validity, and sensitivity in physiological aspects. The EQ-5D-3L is suitable for evaluating the health-related quality of life for the spouses of breast cancer patients. The EQ-5D-3L health utility score was 0.870. The health-related quality of life of spouses in BC patients was poor. The health-related quality of life in spouses of BC patients is equivalent to the disease burden faced by male hypertensive patients and diabetic patients. The health-related quality of life of spouses in BC patients is influenced by social support and cognitive factors for treatment outcomes.

## Data availability statement

The datasets presented in this study can be found in online repositories. The names of the repository/repositories and accession number(s) can be found in the article/supplementary material.

## Ethics statement

The studies involving human participants were reviewed and approved by the ethics research committee of Kunming Medical University. The patients/participants provided their written informed consent to participate in this study.

## Author contributions

L-FS and J-YN conceived and designed the study. L-FS, SH and Y-FL performed the experiments. L-FS, SH and Y-FL wrote the paper. Z-QY, X-WW and J-YN reviewed and edited the manuscript. X-JY collected the patients following data. J-YZ and X-WW performed the data statistical analysis. All authors contributed to the article and approved the submitted version. 

## Funding

Yunnan provincial youth and mid-aged pioneer of science and technology cultivation programme (No.202205AC160059 to X-WW).

## Conflict of interest

The authors declare that the research was conducted in the absence of any commercial or financial relationships that could be construed as a potential conflict of interest.

## Publisher’s note

All claims expressed in this article are solely those of the authors and do not necessarily represent those of their affiliated organizations, or those of the publisher, the editors and the reviewers. Any product that may be evaluated in this article, or claim that may be made by its manufacturer, is not guaranteed or endorsed by the publisher.
